# Quantifying non‐clinical outcomes of ultra‐hypofractionated breast radiotherapy in Western NSW—A narrative review

**DOI:** 10.1002/jmrs.842

**Published:** 2024-12-09

**Authors:** Faith Yeo, Rachael Beldham‐Collins, Paul Roth, Rodney Hammond

**Affiliations:** ^1^ Sydney Medical School University of Sydney Camperdown New South Wales Australia; ^2^ School of Rural Health, Faculty of Medicine and Health University of Sydney 4 Moran Drive Dubbo 2830 New South Wales Australia; ^3^ Central West Cancer Centre Orange Health Service Orange New South Wales Australia; ^4^ Western Cancer Centre Dubbo Health Service Dubbo New South Wales Australia

**Keywords:** Breast cancer, non‐clinical benefits, rural, triple bottom line, ultra‐hypofractionated radiotherapy

## Abstract

Ultra‐Hypofractionated Whole Breast Radiotherapy (U‐WBRT) has been proven to be a viable treatment option for breast cancer patients receiving radiation therapy, however, due to its novelty our understanding of its non‐clinical benefits is still evolving. With increasing U‐WBRT selection during COVID and in rural and regional settings such as the Western New South Wales Local Health District (WNSWLHD), it's important to quantify the savings when compared to other fractionation schedules (e.g. Conventional fractionation (C‐WBRT) involving 25 fractions and Moderate hypofractionation (M‐WBRT) with 15 fractions.) Using literature sourced from Medline, Embase, Pubmed and reports from relevant websites and organisations this narrative review investigates quantifiable methods of assessing non‐clinical benefits of U‐WBRT in rural settings according to the triple bottom line philosophy. This review was able to identify a standard set of quantifiable metrics that can compare the non‐clinical benefits of various fractionation schedules, with relevance to a rural setting. These include: fractionation trends, financial subsidy, average linear accelerator (Linac) minutes, hospital visits, travel time and distance, Linac energy consumption, travel and Linac carbon emissions. By identifying these metrics, non‐clinical benefits between the fractionation schedules can easily be quantified and compared.

## Graphical abstract

Ultra‐Hypofractionated Whole Breast Radiotherapy (U‐WBRT) has been proven to be a viable treatment option for breast cancer patients receiving radiation therapy, however, due to its novelty our understanding of its non‐clinical benefits is still evolving. This review was able to identify a standard set of quantifiable metrics that can compare the non‐clinical benefits of various fractionation schedules, with relevance to a rural setting. By identifying these metrics, non‐clinical benefits between the fractionation schedules can easily be quantified and compared.

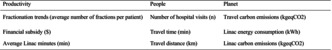

## Introduction

Breast cancer is the most commonly diagnosed cancer among Australian women.^1^ Traditionally, the treatment for early‐stage breast cancer involves breast‐conservation surgery and whole breast radiation therapy (WBRT).^2^, ^3^ Fractionation approaches have shifted from conventional fractionation (C‐WBRT) involving 25 fractions to moderate hypofractionation (M‐WBRT) with 15 fractions to the latest Ultra‐hypofractionation (U‐WBRT) of just 5 fractions.^3^ Studies showed that this U‐WBRT schedule is as effective and safe as the international standard 15‐fraction regimen.^4^ The proportion of early‐stage breast cancer patients in the Western New South Wales Local Health District (WNSWLHD) cancer centres who received hypofractionation regimens from 2018 to 2021^5^, ^6^ has risen. Significant demand has been placed on radiotherapy resources in the region, prompting clinicians to look to U‐WBRT as an effective solution to maximise access to specialised treatment and infrastructure.

With the increasing trend towards U‐WBRT selection by patients and clinicians, it is important to be able to quantify its savings when compared to M‐WBRT and C‐WBRT. To date, there are no articles with a well‐rounded comparison of non‐clinical benefits of U‐WBRT, with many just focusing on a specific topic (e.g. environmental impact or work hours absences^7^). As a starting point, a standardised system of measurement with identified variables is needed to compare savings to other fractionation schedules. To provide structure for the project, the triple bottom line philosophy, a sustainability framework that evaluates overall impact,^8^ was used to investigate the variables used to quantify non‐clinical outcomes. This framework involves the three dimensions of Productivity, People and Planet.^8^ These elements are helpful in visualising and integrating the different aspects of practice in order to examine the full picture of sustainability.^8^ The productivity element examines the profitable benefits of having a shorter fractionation schedule, such as the savings for departmental or governmental costs, resource utilisation and improvement. The people element explores the social impact of U‐WBRT on patients. While the planet element examines how U‐WBRT and the Radiation Therapy (RT) service affect the environment. This study aims to identify a set of metrics that can be used to quantify the non‐clinical benefits of U‐WBRT according to the triple bottom line philosophy in the setting of WNSWLHD. The identified variables can act as a guide for future treatment regimen decisions and future quality improvement in RT facilities, specifically in rural areas.

## Method

### Databases and search strategy

To identify relevant articles, the following electronic databases were searched: Medline, Embase and Pubmed. The search strategy had limits set for English language publications 2010–2024. Combinations of the following search terms (keyword and MeSH) were used: ‘breast cancer’, ‘breast neoplasm’, ‘radiotherapy’, ‘hypofractionated radiotherapy’, ‘conformal radiotherapy’, ‘image‐guided radiotherapy’, ‘cancer radiotherapy’, ‘breast radiotherapy’, ‘adjuvant radiotherapy’, ‘external beam radiotherapy’, ‘radiation dose fractionation’, ‘fractionation’, ‘hypofractionation’, ‘ultra hypofractionation’. When classifying each triple bottom line element, additional keywords were generated and combined. The keywords were derived from previously published literature and in relation to the objectives of this study. Productivity: ‘health care costs’, ‘cost‐benefit analysis’, ‘costs’. People: ‘patient preference’, ‘patient satisfaction’, ‘accessibility’, ‘convenience’. Planet: ‘carbon dioxide emission’, ‘greenhouse effect’, ‘carbon footprint’, ‘environmental sustainability’, ‘sustainability’, ‘environmental impact’, ‘environmental footprint’, ‘life cycle assessment’. Reference lists of all retrieved papers were manually searched to identify any articles not located by the electronic search.

### Study Selection

Information featured in the title, abstract and keywords was assessed to evaluate the article's suitability for inclusion. Where there was inadequate information in the title and abstract to determine suitability, the full paper was retrieved and reviewed. Scientific papers that included primary research investigating breast cancer RT and literature reviews were included, with a focus on non‐clinical benefits and comparisons between the different fractionation schedules (U‐WBRT, M‐WBRT, C‐WBRT). Opinion‐based and editorial publications were excluded.

## Results

**Figure 1:**
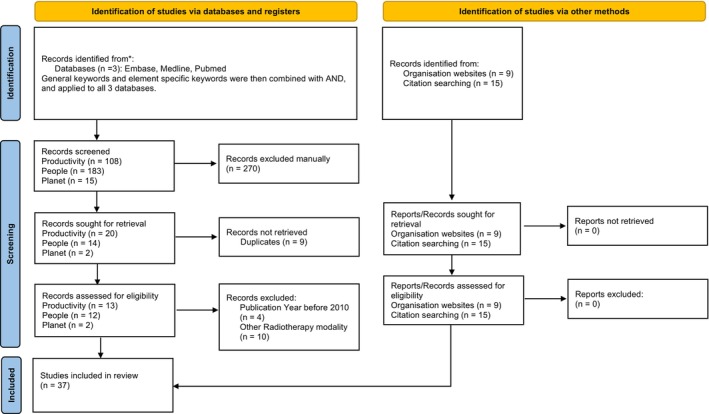
Flow chart depicting the search strategy. A total of 37 articles were included in this review, with 13 articles extracted from the databases search, 15 articles from citation searching and 9 articles from websites and reports. Template from PRISMA.^39^

The results of the literature search were divided into the three dimensions of the triple bottom line: Productivity, People, Planet. Refer to ‘Figure 1’ for the flow chart of the search strategy. Tables 1 and 2 depict the key findings of the articles surveyed and the related elements. A total of 37 articles were included in this review. Overall, 14 articles were identified to discuss productivity elements, 10 articles were related to the people element and 9 articles were associated with the planet elements. Some articles were identified to have overlapping elements, as shown in Table 1.

**Table 1:** Details of relevant primary records extracted from the database and citation searching and included in the study.

Article No.	Study Reference & Journal	Country of study	Study design	Sample (*n*)	Key Findings	Triple bottom line element
1	Dwyer P, et al 2010. Journal of Medical Imaging and Radiation Oncology.^11^	Australia	Retrospective review, quantitative analysis	*n* = 279	Had all eligible patients received M‐WBRT, an extra 14 patients each month could be treated and health care costs would be reduced by 24%.	Productivity, People
2	Batumalai V, et al 2020. Radiotherapy and Oncology.^15^	Australia	Quantitative analysis	*n* = 10,482	Potential cost savings of $13.6 million which would be a 26% reduction in breast RT costs.	Productivity
3	Meaglia I, et al 2019. Radiotherapy and Oncology.^13^	Italy	Quantitative analysis, clinical investigation	*n* = 162	Median 5 days waiting time saved with hypofractionation, and 52 more patients can undergo treatment.	People
4	Bekelman JE, et al 2014. JAMA.^10^	USA	Retrospective, observational cohort study	*n* = 15,643	Hypofractoionation increased by 8.1% (95% CI, 6.0%–10.2%) in 2008 to 21.2% (95% CI, 18.9%–23.6%) in 2013. Difference in total health care expenditures was $8587 (95% CI, $5316–$12,017; *P* < 0.001).	Productivity
5	Busschaert S‐L, et al 2024. Radiotherapy and Oncology.^16^	Belgium	Quantitative analysis, Time‐driven activity‐based costing (TD‐ABC)	*n* = 383	The application of U‐WBRT can permit radiotherapy departments to reduce the use of scarce resources, realise work time and cost savings, increase throughput and reduce waiting times.	Productivity
6	Mortimer JW, et al 2016. Journal of Medical Imaging and Radiation Oncology.^17^	Australia	Quantitative analysis	*n* = 196	Total costs were $5613 AUD per patient for M‐WBRT vs $8272 AUD per patient for C‐WBRT (*P* < 0.001).	Productivity
7	Eckstein J, et al 2022. Cancers.^18^	USA	Protocol development, Qualitative analysis	*n* = 15	Patient travel burden was the factor most strongly impacting radiation oncologists' decision‐making. Relative value unit (RVU) reimbursement and financial toxicity to the patient were reported to be less important in decision‐making.	Productivity
8	Yaremko HL, et al 2021. Current Oncology.^21^	Canada	Quantitative analysis, Cost Minimization Analysis	N/A	Sensitivity analysis shows a maximum per patient cost savings ranging from $474.60 to $508.53 for the FAST‐Forward 1 trial, which translates to an annual savings of $174,700/year locally and $2.06 million/year province‐wide, based on a moderate‐to‐large size department workload.	Productivity
9	Kawaguchi H, et al 2019. Japanese Journal of Clinical Oncology.^24^	Japan	Quantitative and Qualitative analysis	*n* = 426	Reasons for selecting HF‐WBI were as follows: early completion of treatment was more convenient, as long as the two regimens were equivalent in terms of efficacy and side effects (31.8%); work‐related matters (22.7%); advice of a radiation oncologist (15.9%); and difficulty in attending the hospital (15.9%).	People
10	Delaney GP, et al 2016. International Journal of Radiation Oncology*Biology*Physics.^28^	Australia	Quantitative analysis	*n* = 5880	Overall, the use of hypofractionation increased from 37% in 2008 to 48% in 2012 (range, 7%–94% across departments). Treatment facility and the radiation oncologist prescribing the treatment were the strongest independent predictors of hypofractionation.	People
11	Koulis TA, et al 2018. Current Oncology.^29^	Canada	Quantitative analysis	*n* = 4016	Hypofractionated treatment was associated with older age (95% CI: 0.92 to 0.96; *P* < 0.001), and living more than 400 km from a treatment centre (95% CI: 0.16 to 0.86; *P* = 0.02).	People
12	Larios D, et al 2022. International Journal of Radiation Oncology*Biology*Physics.^32^	USA	Quantitative analysis	N/A	CO2 emissions produced from a patient's commute to RT were associated with a 6.5‐fold increase (8484 vs 1302 gCO2) when travelling from a rural area by gas car compared to an urban area via public (metro) transportation.	Planet
13	Gomis Sellés E, et al 2024. Clinical & Translational Oncology.^34^	Spain	Observational evaluation, quantitative analysis	*n* = 1959	Significant reduction in the carbon emissions with U‐WBRT compared to M‐WBRT (26.69 kg vs 57.13 kg, *P* < 0.001).	Planet
14	Freedman Gm 2023. Current Breast Cancer Reports.^3^	USA	Narrative review	N/A	M‐WBRT given Monday through Friday over 3–4 weeks is now standard practice for almost all patients needing postlumpectomy WBRT. For selected patients, there is now an option for ultra‐hypofractionation (U‐WBRT) that further shortens WBRT to as few as 5 treatments in 1 week.	Productivity
15	Murray Brunt A, et al 2020. The Lancet.^4^	UK	Clinical trial	*n* = 4096	26 Gy in five fractions over 1 week is non‐inferior to the standard of 40 Gy in 15 fractions over 3 weeks for local tumour control, and is as safe in terms of normal tissue effects up to 5 years for patients prescribed adjuvant local radiotherapy after primary surgery for early‐stage breast cancer.	Productivity, Planet
16	Aitken K et al 2022. Clinical Oncology.9	UK	Narrative review	N/A	Reducing treatment attendances is appealing to both patients and healthcare providers. During the COVID‐19 pandemic, hypofractionated regimens were rapidly adopted across the UK and internationally.	Productivity, People
17	Butler SM 2017. J Med Radiat Sci.^25^	Australia	Quantitative analysis, cross‐sectional study	N/A	The number of patients who accessed radiation increased from 573 to 667 between 2010 and 2012. The corresponding radiotherapy utilisation (RTU) rates were 29.3% in 2010 and 33.4% in 2012, an improvement of 4.1% (*P* = 0.01, 95% CI: 1–7%). Patients travelled 128.5 km less for treatment in 2012 than in 2010 (338.7 km vs. 210.2 km, CI 111–145.5 km, *P* > 0.0001).	Productivity
18	Howard K, et al 2024. International Journal of Radiation Oncology*Biology*Physics.^22^	Australia	Discrete choice experiment	*n* = 420	Shorter treatment duration, avoiding relocation, fewer local side effects, and less difficulty with daily activities all positively influenced treatment preference.	People
19	Cheung RCM, et al 2023. International Journal of Radiation Oncology*Biology*Physics.^31^	Canada	Quantitative analysis	*n* = 10,175	The use of curative hypofractionated regimens increased from 17% to 27% during the pandemic year. Carbon footprint was reduced by 39% during the pandemic year (1,332,388 kg CO2e) compared with the prepandemic year (2,024,823 kg CO2e).	Productivity, Planet
20	Larios D, et al 2023. International Journal of Radiation Oncology*Biology*Physics.^36^	USA	Life cycle assessment (LCA) methods	*n* = 50	The largest contributors to total emissions in each group were patient and staff transportation (301.8 vs 196.4 kg CO2‐eq, respectively) and LINAC equipment and utilisation (175 vs 55.2 kg CO2‐eq, respectively).	Planet
21	Rivera S, et al 2023. Radiation and Oncology.^35^	France	Quantitative analysis	*n* = 120	The mean travelling distance was 324 km (SE: 42 km) and the mean carbon footprint per patient for the whole 1‐week breast RT was 67 kgeqCO2.	Planet
22	Shah C, et al 2019. International Journal of Radiation Oncology*Biology*Physics.^14^	USA	Quantitative analysis	*n* = 520	Hypofractionated WBRT saved $700 based on direct costs and $1371 including indirect costs.	Productivity
23	Nugent K, et al 2023. The Breast.^20^	Ireland	Clinical implementation, Quantitative analysis	*n* = 135	Delivering U‐WBRT to 135 patients over 6 months saved 21,300 linac minutes and 1485 hospital visits compared to M‐WBRT.	Productivity, People
24	Purden J, et al 2023. Journal of Radiotherapy in Practice.^23^	UK	Quantitative analysis	*n* = 1516	Moving to U‐WBRT saves 20 one‐way trips to the hospital, resulting in an average time saving of 15.9 hours for those travelling by car and 39.3 hours for those travelling by public transport. On average, this reduces carbon dioxide emissions by 91 kg per patient.	People, Planet
25	Tyldesley S, et al 2010. Clinical Oncology.^26^	Canada	Quantitative analysis	*n* = 32,491	The use of radiotherapy fluctuated with place of residence at diagnosis. Breast cancer patients living in rural areas in British Columbia had a 12% lower utilisation of radiotherapy than their counterparts living in urban areas.	People
26	Baldwin LM, et al 2010. Cancer.^27^	USA	Quantitative analysis, logistic regression analyses	*n* = 122,526	Radiation therapy receipt differed more by sociodemographic characteristics (eg, rural patients aged <50 years had a 67.1% receipt rate, whereas those aged ≥80 years had a radiation therapy receipt rate of 29.1%) than rural versus urban residence.	People
27	Anudjo MNK, et al 2023. Radiography.^30^	UK	Systematic literature review	N/A	Three themes emerged as potentially important areas of practice that contribute to environmental footprint: energy consumption and data storage practices; usage of clinical consumables and waste management practices; and activities related to staff and patient travel.	Planet
28	Chuter R, et al 2023. Physica medica.^33^	UK	Quantitative analysis	*n* = 42	Patient travel made up the largest proportion (70%–80%) of the calculated carbon footprint, with linac idle power second with ∼10%.	Planet

Abbreviations: CI, confidence interval; SE, standard error; N/A, not applicable.

**Table 2:** Details of relevant reports extracted from websites and organisations and included in the study.

Report no.	Title (Organisation)	Key findings
1	Breast Cancer in Australia Statistics (Cancer Australia).^1^	Breast cancer statistics in Australia.
2	Hypofractionated radiotherapy for early (operable) breast cancer (Cancer Australia).^2^	Treatment for early‐stage breast cancer, outcomes of WBRT.
3	Reporting for Better Outcomes: Annual statewide report 2022 (Cancer Institute NSW).^5^	Hypofractionation usage for breast cancer in NSW.
4	WNSWLHD Strategic Plan 2020–2025 (NSW Government).^6^	Population characteristics in WNSWLHD.
5	What is Triple Bottom Line (TBL) and Why is it Important? (TechTarget).^8^	Triple bottom line framework definition and application.
6	Breast Hypofractionation (Cancer Institute NSW).^12^	LBVC initiative, objectives and aims.
7	IPTAAS for Patients (NSW Health).^19^	Isolated Patients Travel and Accommodation Assistance Scheme definition, reimbursement eligibility and rates.
8	Net Zero Plan (NSW Government).^37^	Aims of the NSW Government regarding reducing carbon footprint and emissions.
9	Changes to the Medicare Benefits Schedule for radiation therapy items (Australian Government Department of Health and Aged Care).^38^	New MBS schedule for radiation therapy, of items with benefits weighted to reflect service complexity.

### Metrics to measure the impact on the ‘Productivity’

Over the past 15 years, fractionation trends have observed a similar pattern, with shorter treatment schedules increasingly favoured. The transition of C‐WBRT to M‐WBRT occurred during 2010–2020 and M‐WBRT to U‐WBRT from 2020 to present.^3^, ^9^, ^10^ Clinicians and patients continuously preferred the most time and cost‐efficient regimen. Despite the hypothesised savings of U‐WBRT, there is still variation in adopting hypofractionation regimens in Australia.^11^ The Cancer Institute NSW implemented the Leading Better Value Care (LBVC) Breast Cancer Hypofractionation Initiative,^12^ with aims to increase access to hypofractionation and to reduce variation in the use of hypofractionated breast RT. As such, investigating the fractionation trends in WNSWLHD and similar rural areas can be a starting point for meeting these objectives.

As hypofractionated WBRT involves fewer radiation fractions, radiotherapy‐related expenditures and resources would be significantly reduced.^10^, ^13^ These savings apply to both the medical costs (cost incurred by the RT facility and health care system) as well as the non‐medical costs (patient costs).^14^ As expected, with shorter fractionation schedules, medical costs (e.g. cost per fraction, cost per treatment course) decreased.^15^ This is demonstrated by Dwyer et al,^11^ who found that treatment with hypofractionation would have saved $13.6 million when compared to conventional fractionation treatment. Busschaert et al revealed that average work times and costs per patient decrease when the number of fractions is reduced.^16^ Furthermore, a retrospective study of cancer patients in regional NSW, found that M‐WBRT reduced treatment‐related Medicare costs by an average of $2381 (29.3%) per patient when compared with C‐WBRT.^17^ While these studies provide an idea of the cost savings for U‐WBRT, they do not take into account the costs that rural patients have to incur when seeking treatment. Elderly and rural patients face more significant impaired access to RT treatment especially those living further away from a treatment facility, largely due to barriers such as travel and financial difficulties.^18^ With these adversities affecting treatment adherence, it is important to consider the ways patient costs can be compared across the different fractionation schedules and quantify the savings. Shah et al's USA study was able to determine the transportation costs based on the average number of miles travelled round‐trip and the average reimbursement per mile.^14^ For the WNSWLHD context, a similar method can be used, with travel reimbursement costs calculated using the Isolated Patients Travel and Accommodation Assistance Scheme (IPTAAS).^19^ Likewise, similar governmental subsidies can be applied according to each specific rural jurisdiction.

Additionally, with the shorter treatment phase and the associated lower utilisation of the Linear accelerator (Linac), savings in specialist consultation time, technician operator time and machine time can also be appreciated.^17^ Nugent et al quantified Linac capacity and discovered that delivering the U‐WBRT schedule over 6 months saved 21,300 Linac minutes compared to a M‐WBRT schedule.^20^ Furthermore, the hours per patient per treatment course for U‐WBRT (2.58 h) was found to be almost half that of M‐WBRT (5.33 h).^21^ When these fractionation times were translated to human resource hours and wages, the difference between direct per patient human costs for each regimen was substantial.^21^ The reduction in Linac minutes implies U‐WBRT's ability to increase treatment capacity to meet demands, which would be incredibly beneficial, especially in rural areas. Thus, a metric to calculate the average Linac minutes per fraction and by extension the total Linac minutes in each treatment regimen (U‐WBRT, M‐WBRT and C‐WBRT) should be included to quantify and compare the 3 schedules.

### Metrics to measure the impact on the ‘People’

In line with the LBVC objectives^12^ of improving patient outcomes and experiences of care, Howard et al conducted a discrete choice experiment to understand women's preferences for different attributes of RT treatment.^22^ Results indicated that shorter treatment duration and avoiding relocation were some of the factors significantly influencing treatment preference.^22^ This perspective could drive healthcare provision and planning considerations with regard to standard regimens of breast cancer RT treatment. Nugent et al also found that U‐WBRT saved 1485 hospital visits over 6 months compared to M‐WBRT.^20^ Similarly, a study completed in rural Southwest Wales found that moving to U‐WBRT saves 20 one‐way trips to the hospital.^23^ Kawaguchi et al found that 15.9% of sample patients chose hypofractionation and its associated lesser fractions due to difficulty in attending the hospital.^24^ In the context of regional Australia, the number of required patient attendances is of real consequence due to the challenges of travel and financial burden. With only 2 RT facilities in WNSWLHD, patients across the region may have trouble adhering to and attending treatment.^25^ It would be beneficial to compare the number of hospital visits saved to provide a quantifiable means of comparison should shorter fractionation schedules be used.

Previous studies have found that the use of radiotherapy in different jurisdictions varies in relation to geography, with concerns that patients in rural regions encounter worse access to radiotherapy than patients diagnosed in urban regions closer to cancer centres.^26^, ^27^ Specifically, this variation in hypofractionation uptake could be attributed to the travel distance and time that patients have to endure to attend treatment. Delaney et al found that factors that correlated significantly with hypofractionation use were the distance of patient residence from the department, treating facility and radiation oncologist.^28^ From a study specifically looking at the patients in WNSWLHD, distance remains a significant deterrent to accessing radiotherapy services.^25^ Furthermore, it was found that patients who travelled more than 400 km to receive their RT were more likely to receive a hypofractionated schedule due to the shorter treatment course it offers.^29^ Comparing U‐WBRT (5 fractions) to M‐WBRT (15 fractions) and C‐WBRT (25 fractions), patients would be saving 10 and 20 trips to the RT facility respectively, significantly reducing travel time and distance. With kilometres travelled and travel time being reduced by 3 and 5‐fold, this brings implications to patient and clinician preference for treatment due to increased convenience. Purden et al demonstrated this by revealing that U‐WBRT would save an average of 15.9 hours for those travelling by car and 39.3 hours for those travelling by public transport compared to M‐WBRT.^23^ Therefore, it stands to reason that travel time and distance saved by the patient could be baseline metrics used to compare fractionation regimens and guide treatment choice.

### Metrics to measure the impact on the ‘Planet’

Recent evidence suggests that the carbon footprint from healthcare is significant, with approximately 10% being attributed to clinical radiology and radiotherapy waste,^30^ there is a greater focus on strategies to reduce the environmental impact of such treatment. Hypothetically, the shorter fractionation schedule of U‐WBRT and its benefits of reduced travel and total Linac minutes would offer savings in carbon emissions. This is evidenced by the carbon footprint being reduced by 39% as the use of curative hypofractionated regimens increased from 17% to 27% during the pandemic year.^31^ Extending from the issue of travel, increased travel would translate to increased travel CO2 emissions. A study found that CO2 emissions had a 6.5‐fold increase when travelling to a RT facility by car from a rural area compared to via public transportation from an urban area.^32^ Especially in rural areas, where public transport is frequently scarcer, the use of private vehicles to travel is more prominent. Travel was also found to have the most significant impact on overall carbon emissions, making up to 70–80% of the calculated carbon footprint.^33^ As such, reducing the number of fractions and, therefore, visits to the RT facility would potentially result in significant savings of carbon emissions. In support of this, the study by Sellés et al revealed that U‐WBRT saves an average of 144.50 kgCO2 in terms of travel compared to C‐WBRT (83.3% less emissions) and 55.90 kgCO2 compared to M‐WBRT (51.6% less emissions).^34^ Consequently, it can be estimated that a reduction in the number of fractions from 25 (C‐WBRT) or 15 (M‐WBRT) to 5 (U‐WBRT) for breast RT could reduce carbon footprint by a factor 5 or 3 respectively.^35^ Therefore, travel carbon emission is a valuable metric to use as a comparison between the 3 fractionation schedules.

Another large contributor to carbon emissions would be Linac equipment and utilisation.^36^ To calculate Linac carbon emissions, Sellés et al first evaluated the Linac power consumption according to manufacturer product information and found a significant decrease (*P* < 0.001) in the energy consumed in U‐WBRT compared to M‐WBRT (9.24kWh vs. 5.345kWh; 42.1% less).^34^ This is in line with the assumption that with lesser fractions and lesser total Linac minutes (as detailed above), there would be reduced electrical consumption of the Linac machine. Converting the power consumption to carbon emissions produced significant results as well, with U‐WBRT having a reduction of emissions of 49% compared to M‐WBRT.^34^ Another study also found a 3.22‐fold increase in Linac CO2 emissions with M‐WBRT vs U‐WBRT (67 vs 21 kgCO2, *P* < 0.01).^31^ To aid in the NSW Net Zero Plan,^37^ metrics to compare savings of Linac carbon emissions and power consumption should be included.

### Identified metrics

From the evaluated literature, this study was able to ascertain the essential metrics that can provide an effective comparison between the 3 schedules, from a rural perspective (Table 3). These include: fractionation trends (average number of fractions per patient), financial subsidy ($), average Linac minutes (min), number of hospital visits (*n*), travel time (min) and distance (km), travel carbon emissions (kgeqCO2), Linac energy consumption (kWh) and Linac carbon emissions (kgeqCO2).

**Table 3:** Identified metrics according to the triple bottom line elements.

Productivity	People	Planet
Fractionation trends (average number of fractions per patient)	Number of hospital visits (*n*)	Travel carbon emissions (kgeqCO_2_)
Financial subsidy ($)	Travel time (min)	Linac energy consumption (kWh)
Average Linac minutes (min)	Travel distance (km)	Linac carbon emissions (kgeqCO_2_)

### Limitations

Considerations need to be given to other non‐clinical benefits that U‐WBRT has to offer that have not been explored in the review. Due to the limited data and lack of articles comparing these variables, factors such as patient relocation, work days/hours lost, accommodation costs, departmental costs and carbon footprint of consumable single‐use items were not studied. Additionally, as this study is a review of the current literature, there may be other factors that previous studies did not investigate and were, therefore, not analysed by this study. With the exclusion of non‐English language publications, studies offering a different perspective may have also been missed. Further research could be undertaken to investigate these features to provide a well‐rounded view of U‐WBRT.

### Implications for practice

The non‐clinical focus of the identified metrics allows for their use in RT departmental activities such as research, quality improvement, clinical audit, business cases and grant applications. These measures can also be used to advocate for resources and funding from state and federal agencies by indicating or forecasting the improved efficiency, government savings and reduction in the carbon footprint of implementing U‐WBRT at a treatment centre.

The metrics can be applied to other treatment sites and schedules and any rural or regional jurisdiction. Application of the measures will require access to electronic medical records in treatment centres and clinicians need to ensure local clinical governance guidelines are followed when accessing such patient data. If the data are to be used in the research setting, the appropriate quality assurance and/or research ethics processes will need to be completed. However, data accuracy and completeness may prove to be an issue when accessing these metrics. For example, RT facilities may not keep records of patient travel and accommodation during treatment and patient addresses may not be kept up to date. For application to a metropolitan setting, the metrics relating to travel would be less relevant due to the increased accessibility of RT facilities and public transport. However, other metrics such as fractionation trends, number of hospital visits, average Linac minutes, Linac energy consumption and carbon emissions are still applicable. As such, comparisons of metropolitan to rural and regional data can still be made specifically for these metrics, thereby assisting with highlighting important issues regarding equity of care and access for cancer patients.

It is worth noting that despite the prospected savings of U‐WBRT, other resource demands such as RT simulation and planning still remain. Recent changes to the Australian Medicare Benefits Schedule (MBS) for radiotherapy acknowledge this ongoing demand with an increased weighting for these items to reflect their increasing complexity and use in modern clinical practice.^38^ Despite this, for treatment, the MBS has a pay‐per‐fraction approach which could be a barrier for the adoption of U‐WBRT considering the lesser fractions that accompany it. Moving to a pay‐per‐course (or similar model) may be a solution to overcome this, however, the implications and risks would need appropriate clinical investigation and assessment before such implementation. The improved treatment capacity that U‐WBRT generates has valuable implications and could potentially allow RT departments to repurpose staff and resources to assist in other areas. The increased capacity could also aid in meeting future treatment utilisation growth, especially in rural centres, without the need for capital expansion or budget allocation.

## Conclusion

In conclusion, this article has explored the non‐clinical benefits of shorter fractionation schedules and identified several quantifiable metrics that can be used as a template to compare the savings of different fractionation schedules. In line with the LBVC objectives,^12^ this study offers a better understanding of the variation in breast RT schedules and how U‐WBRT is able to deliver assistance for the demand of services. With the outcomes from this project, quantifiable savings of U‐WBRT can easily be identified and compared to M‐WBRT and C‐WBRT, thus guiding clinician and patient choices of treatment. Once established, strategies to better utilise resources can be developed to maximise the non‐clinical benefits of U‐WBRT and address the variation in the use of hypofractionated radiotherapy in NSW.^12^ These metrics could also possibly be extended to other treatment body sites, prompting further research in these areas.

## Conflict of Interest

The authors declare no conflict of interest. Due to the nature of this narrative review, no local ethics approval was required.

## Data Availability

Data sharing not applicable to this article as no datasets were generated or analysed during the current study.
